# Current practices in cancer pain management in Asia: a survey of patients and physicians across 10 countries

**DOI:** 10.1002/cam4.471

**Published:** 2015-04-27

**Authors:** Yong-Chul Kim, Jin Seok Ahn, Maria Minerva P Calimag, Ta Chung Chao, Kok Yuen Ho, Lye Mun Tho, Zhong-Jun Xia, Lois Ward, Hanlim Moon, Abhishek Bhagat

**Affiliations:** 1 Department of Anesthesiology and Pain Medicine, Seoul National University School of MedicineSeoul, Korea; 2 Department of Medicine, Samsung Medical Center, Sungkyunkwan University School of MedicineSeoul, Korea; 3 Departments of Pharmacology, Clinical Epidemiology and Anesthesiology, University of Santo Tomas Faculty of Medicine and Surgery and the UST HospitalManila, Philippines; 4 Institute of Clinical Medicine, School of Medicine, National Yang-Ming UniversityTaipei, Taiwan, China; 5 Division of Hematology and Oncology, Department of Medicine, Taipei Veterans General HospitalTaipei, Taiwan, China; 6 Pain Management Service, Raffles HospitalSingapore, Singapore; 7 Department of Clinical Oncology, Beacon International Specialist CentreSelangor, Malaysia; 8 Sun Yat-Sen University Cancer CenterGuangzhou, China; 9 Mundipharma Research LtdCambridge, United Kingdom; 10 Mundipharma Pte LtdSingapore, Singapore

**Keywords:** Cancer pain, pain management survey

## Abstract

In order to implement more effective policies for cancer pain management, a better understanding of current practices is needed. Physicians managing cancer pain and patients experiencing cancer pain were randomly surveyed across 10 Asian countries to assess attitudes and perceptions toward cancer pain management. A total of 463 physicians (77.3% oncologists) with a median experience of 13 years were included. Medical school training on opioid use was considered inadequate by 30.5% of physicians and 55.9% indicated ≤10 h of continuing medical education (CME). Of the 1190 patients included, 1026 reported moderate-to-severe pain (median duration, 12 months). Discordance was observed between physician and patient outcomes on pain assessment with 88.3% of physicians reporting pain quantification, while 49.5% of patients claimed that no scale was used. Inadequate assessment of pain was recognized as a barrier to therapy optimization by 49.7% of physicians. Additional barriers identified were patients’ reluctance owing to fear of addiction (67.2%) and adverse events (65.0%), patients’ reluctance to report pain (52.5%), excessive regulations (48.0%) and reluctance to prescribe opioids (42.8%). Opioid use was confirmed only in 53.2% (286/538) of patients remembering their medication. Pain affected the activities of daily living for 81.3% of patients. These findings highlight the need for better training and CME opportunities for cancer pain management in Asia. Collaborative efforts between physicians, patients, policy makers, and related parties may assist in overcoming the barriers identified. Addressing the opioid stigma and enhancing awareness is vital to improving current standards of patient care.

## Introduction

The incidence of cancer in Asia is expected to reach 10.6 million by the year 2030 1. Pain in cancer patients is a major medical issue with a pooled prevalence rate >50% for all cancer types 2. In advanced disease stages, 70–80% of cancer patients suffer from uncontrolled pain of moderate-to-severe intensity 3. This issue has become of increasing concern in Asia, particularly in low- and middle-income countries where 70% of cases are diagnosed in locally advanced or advanced clinical stages, with overall 5-year survival rates of <50% 3,4. In terminally ill patients, pain relief and other palliative care is recognized as the primary goal of treatment 5. Despite the publication of international guidelines, there persist significant inadequacies in terms of cancer pain management across the globe 6–8. The prevalence of under-treated cancer pain in Asia has been reported to range from 27% to 79%, with a weighted mean of 59.1%, compared with mean values of 39.1% and 40.3% in North America and Europe, respectively 3.

The consequences of inadequate pain management are exacerbated in developing Asian countries due to the socioeconomic burden associated with life-threatening disease conditions. Inadequate assessment of pain, opioid access and regulations, and stigmas associated with opioid use are significant barriers to optimal pain management 9. Although opioid therapy is a first-line treatment option for the management of moderate-to-severe pain, several regional surveys in Asia have shown a reluctance to prescribe opioids to cancer patients and below average rates for regular opioid use 10–12. In some Asian countries, accessibility to opioids is severely restricted, leaving little therapeutic alternatives for the treatment of serious pain. Although opioid analgesics are readily available in some economically developed countries such as Singapore, consumption is reported to be limited due to a variety of factors 13.

The ability to undertake informed policy decisions will require relevant data regarding cancer pain management practices in Asia. This cross-sectional ACHEON survey was designed to evaluate the current status of cancer pain management and includes data from 460 physicians in cancer practice and 1180 cancer patients from 10 countries/regions in Asia. To the best of our knowledge, this is the largest survey of its kind to evaluate the attitudes and perceptions of physicians treating cancer pain, as well as pain severity and quality of life (QoL) in cancer patients.

## Methods

### Survey design

Physicians and patients were recruited from 10 countries/regions across Asia, including China (CN), Hong Kong (HK), Indonesia (INDO), the Republic of Korea (SK), Malaysia (MY), Philippines (PH), Singapore (SG), Taiwan (TW), Thailand (TH), and Vietnam (VN), and surveyed anonymously over a period of 4 months (September–December 2013). ACHEON was a questionnaire-based survey and required no other intervention involving the participants. In some cases, respondents included noninstitutionally affiliated patients experiencing cancer pain, as well as physicians operating private practices. An ethical review fulfilling the function of an IRB (Institutional Review Board) was therefore conducted, and the study design and conduct was reviewed by a group of 16 external experts from various countries in Asia. The survey was additionally conducted in accordance with the European Society for Opinion and Market Research (ESOMAR) code of conduct for market research studies 14. All physicians and patients participating in the survey were guaranteed confidentiality and anonymity. Surveyors were required to conform to all national and international laws while ensuring that the rights of all respondents were respected and data were not used for any unauthorized purposes. The respondents’ cooperation was voluntary, with all survey activities carried out in a transparent and objective manner as outlined in the protocol.

#### Objectives

The primary objective of the present study was to assess the extent and impact of cancer pain on patients’ lives, (in terms of activities of daily living, aspects of QoL, and employment) their levels of satisfaction with treatment, and the perceived efficacy of their prescribed pain medication. We also sought to assess the attitudes and perceptions of physicians and patients toward cancer pain management in Asia and identify potential barriers to opioid prescription.

#### Selection of physicians

Physicians were selected according to eligibility criteria and included those involved in managing cancer pain, those involved in the direct care of patients with cancer pain and those willing to participate in all aspects of the study. They were randomly selected from databases of medical associations, national registries, official societies, and other organizations. Physicians who were employed by or affiliated with a pharmaceutical company were excluded from the survey.

#### Selection of patients

Patients aged ≥18 years with a documented history of cancer pain in the preceding month were randomly contacted through patients’ associations, doctor referrals, hospital intercepts, online panels, patient referrals, door-to-door recruitment, or phone book recruitment. Patients who were employees or related to employees of any health-care, pharmaceutical, advertising or market research companies, or those involved in any other pain management studies were excluded.

#### Questionnaires

The questionnaires for the physician and patient surveys were developed by a steering committee of 16 pain management specialists, representing most of the participating countries. A consensus questionnaire was created, which was then translated into the local languages of the participating countries.

The questions for physicians were grouped into broad categories which included demographic data (including medical expertize), education and training, screening and assessment of pain, and pain management practices. Accordingly, the questions for patients were focused on demographic data, screening for pain and pain assessment, perceived doctors’ attitudes toward pain, effect of pain on QoL and activities of daily living (including employment), and treatment of pain.

#### Survey methods

Informed consent from the physicians was obtained following initial contact via telephone or face-to-face meetings in which a description of the study was provided. Subsequently, interviews were scheduled via telephone or face-to-face meetings. Patients meeting the inclusion criteria were administered a paper- or a web-based secure link questionnaire for self-completion, which also included a patient disclosure and informed consent section.

### Data analysis

Physician profiling was conducted on the basis of questions related to age, sex, years in practice, specialization, adequacy of training in medical school, and residency. Numeric rating scales (NRS) from 0 to 10 were used to evaluate attitudes and clinical practice patterns including screening, assessment, and optimization of cancer pain management with an emphasis on opioid analgesics. Participants had the option of selecting more than one response in some categories. Depending on the nature of the statement, an NRS score >5 (high) represented adequacy or agreement, an NRS score of 5 was considered neutral and NRS scores <5 (low) indicated inadequacy or disagreement. These categorized scores were reported as a percentage of all respondents and median (interquartile range, IQR) scores were calculated where applicable. The questionnaire for patients was designed to assess the severity of pain experienced using the verbal 11-point Box Scale (BS-11) pain scale, perceived attitudes toward pain, satisfaction with treatment and QoL. It included statements with “yes” or “no” responses, or a 5-point Likert rating scale from “completely agree” to “completely disagree.”

## Results

### General characteristics of physicians and patients

Responses from 463 physicians managing cancer pain and 1190 patients experiencing cancer pain were analyzed (demographic characteristics are presented in Tables1 and 2). The physicians, 77.3% of whom were oncologists, had a median experience of 13 years (IQR, 12) in clinical practice. The majority of patients included in the analysis (86.2%, *n* = 1026) reported suffering from moderate-to-severe pain (BS-11 median [IQR] score 6.0 [3.0]) for a median duration of 12 (IQR, 19) months.

**Table 1:** Demographic characteristics of physicians (*n* = 463)

Parameters	
Age, years	
Median (IQR)	42 (13)
Gender	*n* (%)
Female	150 (32.4)
Male	313 (67.6)
Country/region	*n* (%)
China	100 (21.6)
Republic of Korea	75 (16.2)
Vietnam	51 (11.0)
Philippines	50 (10.8)
Taiwan	50 (10.8)
Indonesia	30 (6.5)
Thailand	30 (6.5)
Malaysia	30 (6.5)
Singapore	30 (6.5)
Hong Kong	17 (3.6)
Years in clinical practice	*n* (%)
1–5	49 (10.6)
6–10	119 (25.7)
11–15	113 (24.4)
16–20	87 (18.8)
>20	95 (20.5)
Area of expertize	*n* (%)
Medical, surgical or hemato-oncology	358 (77.3)
Anesthesiology	52 (11.2)
Pain management	44 (9.5)
Other	9 (1.9)

IQR, interquartile range.

**Table 2:** Demographic characteristics and pain profiles of patients (*n* = 463)

Parameters	
Age, years	
Median (IQR)	53 (17)
Gender	*n* (%)
Female	805 (67.7)
Male	385 (32.3)
Country/region	*n* (%)
China	250 (21.0)
Republic of Korea	150 (12.6)
Philippines	125 (10.5)
Malaysia	102 (8.6)
Hong Kong	100 (8.4)
Indonesia	100 (8.4)
Thailand	100 (8.4)
Vietnam	100 (8.4)
Singapore	88 (7.4)
Taiwan	75 (6.3)
BS-11 pain score	*n* (%)
Severe (7–10)	516 (43.3)
Moderate (4–6)	510 (42.9)
Mild (0–3)	164 (13.8)
BS-11 pain score	
Median (IQR)	6.0 (3.0)
Duration of pain	*n* (%)
More than 1 year	463 (38.9)
<6 months to 1 year	325 (27.3)
3–6 months	297 (25.0)
Less than 3 months	105 (8.8)
Duration of pain, months	
Median (IQR)	12 (19)

IQR, interquartile range.

### Education and training

Medical school training on cancer pain management and opioid use was considered inadequate by 30.5% (*n* = 141/463) of physicians. Notably, 55.9% (*n* = 259) of physicians reported ≤10 h of continuing medical education (CME) training on cancer pain management in the preceding year.

### Clinical practice: screening and assessment of cancer pain

**Figure 1:**
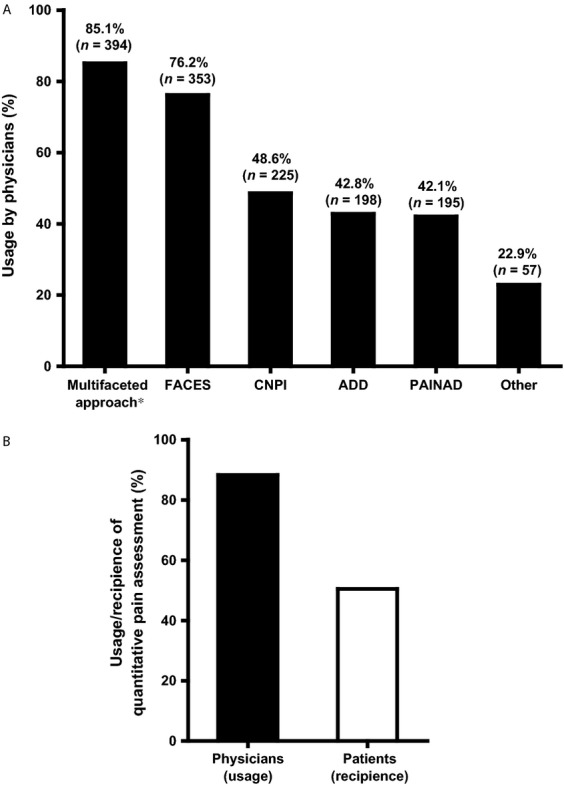
Pain assessment practices by physicians (*n* = 463). (A) Pain assessment tools most commonly employed by physicians (*n* = 463). *Direct observation, family/caregiver input. Respondents had the option to select more than 1 response. ADD, Assessment of Discomfort in Dementia Protocol; CNPI, Checklist of Nonverbal Pain Indicators; PAIN AD, Pain Assessment in Advanced Dementia Scale. (B) Number of respondents confirming quantitative pain assessment in practice (*n* = 463 for physicians, *n* = 1190 for patients). Question in physicians’ survey—“Are all patients who present with pain routinely assessed to quantify pain using a pain scale?” Question in patients’ survey—“Does your doctor always use a pain scale?”

The majority of physicians stated that they assess patients routinely to characterize (90.5%, *n* = 419; median NRS score 9.0 [IQR, 3.0]) and quantify (88.3%, *n* = 409; median NRS score 8.0 [IQR 3.0]) pain. A NRS such as the visual analog scale (VAS), BS-11 pain scale, the FACES or verbal questionnaire (0–10) rating scale were used for pain quantification by 86.8% (*n* = 402) of physicians. The proportional usage of these pain assessment tools is presented in Figure1A.

Results from the patients’ survey indicated that most patients (83.2%, *n* = 990/1190) were asked about pain by their doctors. However, when patients were asked if pain was assessed quantitatively, only 50.5% (*n* = 601) confirmed that it was conducted (Fig.1B). This was also recognized as a barrier to optimizing pain management by 49.7% (*n* = 230) of physicians as “inadequate assessment of pain” (Table3).

**Table 3:** Physicians’ perceived barriers to optimizing cancer pain management (*n *=* *463)

Potential barrier	*n* (%) (NRS score >5)	Median (IQR)
Patient’s reluctance to take opioids due to fear of addiction	311 (67.2)	7.0 (3.0)
Patient’s reluctance to take opioids due to fear of adverse events	301 (65.0)	7.0 (3.0)
Patient’s reluctance to report pain	243 (52.5)	6.0 (5.0)
Inadequate assessment of pain by physicians and/or nurses	230 (49.7)	5.0 (5.0)
Lack of available pain management or palliative care services	226 (48.8)	5.0 (5.0)
Excessive regulation of opioid drugs	222 (48.0)	5.0 (5.0)
Physician’s reluctance to prescribe opioids	198 (42.8)	5.0 (4.0)
Patient’s inability to pay for interventional analgesics/pharmacotherapy/opioid analgesics	173 (37.4)	5.0 (5.0)

An NRS scoring system from 0 to 10 was used to evaluate physicians’ responses. NRS, numeric rating scale; IQR, interquartile range.

**Figure 2:**
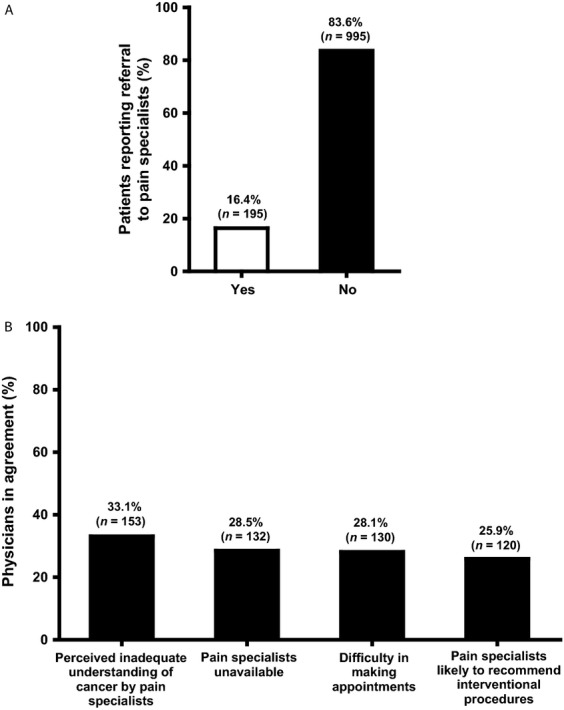
Referrals to pain specialists in clinical practice. (A) Proportion of patients referred to pain specialists (*n* = 1190). (B) Top reasons cited by physicians for not referring patients to pain specialists (*n* = 463). Respondents had the option to select more than one response.

Only 5.9% (*n* = 70) of patients reported that pain specialists were primarily involved in managing their pain, while the majority of patients had never been referred to a pain clinic or pain specialist (Fig.2A). Physicians’ opinions varied with regard to the possible reasons for this observation (Fig.2B), and some regional difference was noted ranging from 59% agreement to “Perceived inadequate understanding of cancer by pain specialists” by HK respondents compared to only 14% of VN respondents. The concern that pain specialists were likely to recommend interventional procedures was generally low, with the exception of INDO, where the response rate was 83%.

Amongst patients who were unemployed, two in five stopped work due to their cancer pain, with the highest proportion of patients who had stopped work due to their pain in TH and the lowest in INDO.

### Clinical practice: management of cancer pain

Overall, physicians expressed unanimity on the effectiveness of opioid use in the management of cancer pain and exhibited a good understanding of dosing regimens. International guidelines were considered important by 56.4% (*n* = 261) of physicians followed by country-specific (24.8%, *n* = 115) and hospital/practice-specific (18.8%, *n* = 87) guidelines. The majority of physicians (83.8%, *n* = 388) agreed that opioid therapy should be first-line therapy in cancer patients with moderate-to-severe pain. A total of 1056 of 1190 patients were currently being treated for pain. However, opioid use was confirmed only in 53.2% of patients (286/538), for those who could remember the type of medication prescribed.

### Barriers to optimization of therapy

Physicians perceived that patient-related factors such as reluctance due to fear of addiction (67.2%, *n* = 311), fear of adverse events (65.0%, *n* = 301), and reluctance to report pain (52.5%, *n* = 243) were important barriers. Excessive regulatory barriers to the use of opioids were identified as a problem by 48.0% (*n* = 222) of physicians (Table3). The concern that pain specialists were likely to recommend interventional procedures was generally low, with the exception of IN, where the response rate was 83%.

### Effects of pain on QoL and activities of daily living

The effects of pain intensity on QoL were assessed by 93.1% (*n* *=* 431) of physicians, however, 78.8% (*n* = 365) of physicians noted discordance between the pain levels they assessed versus the pain experienced by their patients.

A reflection of this observed discordance was also noted in the results from the patients’ survey. In all, 66.6% (*n* = 703) of patients reported that they were satisfied with their treatment and 71.9% (*n* = 759) perceived their medication to be efficacious. However, 81.3% (*n* = 967) of patients cited that chronic cancer pain affected their activities of daily living (Table4).

**Table 4:** Effects of cancer pain on patients’ quality of life (QoL, *n* = 1190)

Effects of cancer pain on QoL	*n* (%) of respondents in agreement
Cancer pain affects patient’s activities of daily living	967 (81.3)
Aspects of daily life affected (*n* = 967)	
Pain affects sleeping patterns	831 (85.9)
Pain affects concentration and focus	841 (87.0)
Pain causes too much reliance on other people	642 (66.4)
Overall QoL is good	328 (33.9)

In a striking observation, it was noted that 77.6% (*n* = 923) of patients were unemployed. Of these, 41.8% had discontinued work due to chronic cancer pain. Additionally, among the patients that were employed, 69.7% (186 of 267) claimed that pain impacted their performance at work.

## Discussion

Despite improving standards of treatment, a diagnosis of cancer invariably has an enormous impact on the lives of its victims. While treatment efficacy and overall survival remain the core focal points of therapeutic efforts, QoL for cancer patients must also remain a central priority if the alleviation of suffering is to be maximized. The side effects of chemotherapy and other treatment approaches often take center stage in public discussions on patient burden, and can eclipse the issue of cancer pain experienced in daily life. In order to better address the full spectrum of patient needs, a comprehensive understanding of the degree of pain experienced and the extent of impact on the lives of patients is critically needed. Social stigmas toward pain and its treatment are also prevalent in many Asian countries and further complicate the issue. We sought to investigate these issues with a large multi-country survey to gain a broader insight into cancer pain practices in the region.

To date, the ACHEON survey is the largest survey conducted to elucidate the attitudes and perceptions of both physicians and patients on pain management in Asia. We recruited 463 physicians, most of whom were oncologists, and 1190 cancer patients who had been suffering from pain for a median duration of 12 months. Overall observations indicate that physicians possess a generally adequate awareness of cancer pain management practices and agree on the effectiveness of opioid use; however, the prescribing of opioids remains inadequate. The ACHEON survey highlighted several patient-, physician-, and regulatory system-related barriers that could be potential contributors to the gap between existing treatment guidelines and real world clinical practice. This is substantiated by other studies highlighting potential barriers to opioid prescriptions among physicians in Asia 13,15–18.

More than half of all physicians indicated that they had received ≤10 h of CME training in the past year, while 30.5% of physicians considered their medical school training on opioid use as inadequate. A previous study involving Chinese physicians indicated that a lack of knowledge and misconceptions played a vital role in impeding morphine use in clinical practice 18. Other surveys from China, Malaysia, the Republic of Korea, and Thailand have reported similar widespread unavailability of satisfactory training 11,19–21. Adequate education for pain management may bolster confidence in prescribing complex therapies while cultivating improved pain assessment practices. Furthermore, it seems likely that many patients would significantly benefit from referral to specialist pain management centers. A change in management practices may be required on the part of physicians treating pain, as it was noted that 83.6% of patients were never referred to a pain clinic.

Inadequate pain assessment was identified as a barrier to optimal therapy, which is in agreement with previous observations indicating a substantial lack of objective pain assessment practices among Asian physicians 9,10,19. Although the evaluation of pain is generally routine, many physicians feel that there is discordance between their own assessment and the actual levels of pain experienced by their patients. There was a discrepancy between physician- and patient-reported procedures for pain assessment; 88.3% of physicians indicated pain quantification was conducted using a pain scale, whereas 49.5% of patients claimed that their pain assessment was primarily subjective without the use of any pain scale.

Medical opioid usage in Asia is lagging behind rates seen in North America and some European nations 6. As evidenced in a meta-analysis by Chen et al., barriers to opioid use perceived by Asian patients using the Barriers Questionnaire were significantly higher than for Western patients (*P* < 0.001) 22. The majority of patients surveyed were suffering from moderate-to-severe pain for more than 12 months, yet reported satisfaction with treatment and perceived their pain medication to be efficacious. This could be indicative of general misconceptions among Asian patients regarding pain alleviation, as cultural taboos sometimes discourage levels of outspokenness accepted by their Western counterparts 23. It has been established that Asian cancer patients believe that pain associated with cancer is an inevitable natural consequence of the disease and can be overcome by positive thinking rather than treatment 23. Indeed, in this survey, physicians perceived the reluctance of patients’ to properly report pain as one of the major barriers to optimal pain management. In accordance with previous studies 9,15–18, patients’ reluctance to accept opioid therapy possibly due to fears of addiction and adverse effects was a primary constraint faced by physicians. Approximately half of the physicians surveyed indicated that regulatory policies were a barrier to prescribing opioids. Striking a balance between regulatory issues and provision of adequate care may be a daunting task for physicians, however, it is necessary to improve the current status of care offered to terminally ill patients. Increasing patient awareness through counseling and education on opioids is also vital to address stigmas associated with opioid use. Previously, follow-up studies in patients using opioid analgesics have reported that the potential for abuse is low when used for the treatment of cancer pain 24,25. Nonetheless, it is imperative that physicians adhere to best prescribing practices that are predisposed toward prevention of such abuse.

In this analysis, a profound impact of pain was apparent on the physical, social, and economic well-being of patients and their overall QoL. Importantly, chronic pain was identified as a major cause of unemployment and impacted work performance for many patients. This has significant negative implications for the entire region, which includes several developing economies.

For the responses from the patients surveyed, we identified some contrasts between the countries and regions (with IQR values larger than 3.0). While most patients reported being satisfied with the way their physicians treat their pain, three-quarters still felt that their doctors would rather treat the cancer rather than the pain and this was most evident in PH, followed by CN, TW and VN. There was also a notably large IQR score of 6.0 regarding the type of pain scale used (with the biggest discrepancies coming from SG and Thailand). When queried as to the reason why patients thought they were not being referred to pain specialists, only 5% of Chinese respondents answered “because they are not available” compared to 66% in the Philippines, indicating a wide discrepancy between these two countries in terms of the availability of these specialists.

Overall, more than four in five patients saw the doctor managing their cancer pain at least once a month. This differed significantly in HK & MY, where only half of all patients reported seeing their doctor as frequently. Half of all patients said their doctor used a pain scale to assess their pain, with HK, CN & SK patients reporting the highest incidence & INDO & TH reporting the lowest. Three-quarters of doctors explained that cancer pain can be controlled, although this was considerably lower in Thailand.

Aside from the mindset of only taking pain medication when absolutely necessary, key reasons for not being treated for pain were cost concerns (PH, VN) and concerns over side effects of pain medication (MY, CN, SK). Over three-quarters of patients interviewed were not currently employed, with the highest employment seen in HK, INDO, MY, and SG. However, whether this was solely due to their cancer pain is difficult to determine.

Global health advocacy groups have reached the consensus that cancer pain relief should be recognized as a human right 6. It has been established that pharmacotherapy can provide satisfactory pain alleviation in 70–90% of patients if treated with an appropriate and timely regimen 2. In order to support this initiative, physicians and patients in Asia must be equipped with effective tools for pain management. Health care professionals may benefit extensively from guidance on comprehensive and customized pain assessments to bridge the gap between professional assessments and the true extent of pain experienced by patients. The implementation of country-based pain management and medical education programs has shown positive long-term benefits in reducing pain intensity 26,27.

As with all questionnaire-based surveys of this kind, our findings were subject to certain limitations due to the qualitative design. Country-dependent discrepancies in recruitment methods cannot be ruled out, and the survey was designed to encompass various scenarios of clinical practice, leading to some restriction in choice of responses. Furthermore, comorbidities were not accounted for, which may have impacted negative perceptions. Nevertheless, we believe these results to be of significant value in that they provide a snapshot of real-life scenarios in clinical practice, and may assist in delineating theoretical assumptions about cancer pain.

## Conclusions

The ACHEON survey results highlight a number of barriers that hinder effective opioid use, as well as the need for better training and CME opportunities for pain management. Inadequate pain assessment practices associated with suboptimal pain management are likely to be reducing QoL for a large number of cancer patients. Addressing the stigmas surrounding opioid use and enhancing awareness is also essential to improve current standards of patient care.
